# Challenges on the development of a pseudotyping assay for Zika glycoproteins

**DOI:** 10.1099/jmm.0.001413

**Published:** 2021-09-09

**Authors:** Fernando Ruiz-Jiménez, Jose Humberto Pérez-Olais, Chidinma Raymond, Barnabas J King, C. Patrick McClure, Richard A. Urbanowicz, Jonathan K. Ball

**Affiliations:** ^1^​ School of Life Sciences, The University of Nottingham, Nottingham, UK; ^2^​ Department of Natural Sciences, Autonomous Metropolitan University, Mexico City, Mexico; ^3^​ Department of Infection Biology and Microbiomes, Institute of Infection, Veterinary and Ecological Sciences, University of Liverpool, Liverpool, UK

**Keywords:** glycoprotein, pseudotype, Zika virus

## Abstract

**Introduction:**

Zika virus (ZIKV) emerged as a public health concern on the American continent during late 2015. As the number of infected grew so did the concerns about its capability to cause long-term damage especially with the appearance of the congenital Zika syndrome (CZS). Proteins from the TAM family of receptor tyrosine kinases (RTKs) were proposed as the cellular receptors, however, due to the ability of the virus to infect a variety of cell lines different strategies to elucidate the tropism of the virus should be investigated.

**Hypothesis:**

Pseudotyping is a powerful tool to interrogate the ability of the glycoprotein (GP) to permit entry of viruses.

**Aim:**

We aimed to establish a highly tractable pseudotype model using lenti- and retro-viral backbones to investigate the entry pathway of ZIKV.

**Methodology:**

We used different glycoprotein constructs and different lenti- or retro-viral backbones, in a matrix of ratios to investigate production of proteins and functional pseudotypes.

**Results:**

Varying the ratio of backbone and glycoprotein plasmids did not yield infectious pseudotypes. Moreover, the supplementation of the ZIKV protease or the substitution of the backbone had no positive impact on the infectivity. We showed production of the proteins in producer cells implying the lack of infectious pseudotypes is due to a lack of successful glycoprotein incorporation, rather than lack of protein production.

**Conclusion:**

In line with other reports, we were unable to successfully produce infectious pseudotypes using the variety of methods described. Other strategies may be more suitable in the development of an efficient pseudotype model for ZIKV and other flaviviruses.

## Introduction

### ZIKV overview

Zika virus (ZIKV) is a member of the Flavivirus genus of the *Flaviviridae* family. Flaviviruses are enveloped viruses with a single strand of positive-sense RNA with a size of ~10.8 kb. The genome is associated with the capsid protein (C), which is surrounded by a lipid membrane associated with the envelope (E) protein and the membrane (M) protein in the mature virion. The genome contains an ~100 nt 5′ untranslated region (UTR), a single open reading frame of ~10 kb, and an ~420 nt 3′ UTR. A single ORF codifies for a single polyprotein that is cleaved by cellular or viral proteases to produce three structural proteins (C, PrM/M, E), and seven non-structural (NS1, NS2A, NS2B, NS3, NS4A, NS4B, NS5) [1, 2].

### ZIKV expansion

ZIKV has emerged as a new infection across the American continent. *Aedes Aegypti* is the primary vector for ZIKV but is also implicated in the spread of other flaviviruses such as Yellow Fever (YF), Dengue (DENV), Chikungunya (CHIKV) and West Nile (WNV). Other species of Aedes capable of spreading ZIKV are *A. albopictus, A. hensilli* (Yap Islands) and *Aedes polyniensis* (French Polynesia) [3].

ZIKV was first isolated in 1947 in the Zika forest near Entebbe, Uganda, from the serum of a sentinel macaque (strain MR766) [1]. The virus was recognized as a human disease-causing pathogen when three people fell ill in Nigeria [4]. After the discovery ZIKV infections were occasionally reported across Africa, Malaysia and Indonesia [5]. During 2007, there was an outbreak in the Yap Islands followed by a more significant outbreak in French Polynesia and Easter Island during 2013–2014. The outbreak in French Polynesia has a role in the passage of the virus to the Americas because the seroprevalence of ZIKV within the island increased dramatically from 0.8 % in October 2013 to 66 % in December 2014 [6]. The epidemic then spread to Easter Island and the Cook Islands reaching continental America in 2015 [3]. As of June 2021, there have been a total of 882 877 reported cases (autochthonous suspected or confirmed) with 253 109 laboratory confirmed cases of the virus since 2015 in the Americas [7].

### Impact of ZIKV infection during neural development

ZIKV infection produces a mild febrile disease with a spectrum of symptoms such as rash, fever, arthralgia and conjunctivitis. However, the infection may cause severe clinical manifestations in new borne from infected mothers known as congenital Zika syndrome (CZS), with microcephaly the most extreme manifestation. CZS comprises a range of congenital central neural system disorders with different clinical signs such as slight cerebral volume reduction, severe cerebral volume reduction and obstructive hydrocephalus, calcifications in the basal ganglia and thalamus, calcifications in the brainstem and cerebellum and cerebellar hypoplasia affecting the vermis and hemispheres [8].

ZIKV shows tropism for radial glial progenitor (RGP) cells; these cells are highly polarized and elongated, covering all the layers of the newly formed neocortex. It has been recently demonstrated that the genetic alterations that affect the survival or the proliferation of the RGP lead to microcephaly [9]. Due to its impact during gestation and the possible development of microcephaly during infection, it is crucial to dissect the initial entry mechanisms of the virus. The viral proteins M and E play a pivotal role during the receptor binding and internalization steps, due to the direct interaction with the host receptor [10]. Axl has been proposed as one of the receptors for ZIKV, showing high expression in radial glial cells and during the middle stages of neurogenesis, Axl is highly expressed on the border of the lateral ventricle and in the outer subventricular zone (OSVZ) [11]. However, the ablation of Axl in cultured neural progenitor cells did not protect the cells against ZIKV infection, suggesting a degree of redundancy in the receptors required for entry [12]. To further investigate the interplay between the viral glycoprotein and the receptors or cellular factors required for efficient infection, different models can be used; viral pseudotypes are one of the safest and more versatile [13].

Pseudotyping is one of the most useful tools to investigate the glycoprotein-dependent entry pathway of different viruses. Pseudotypes are viral particles that are comprised of a vector, most frequently lentiviral vectors such as Human Immunodeficiency Virus type 1 (HIV-1) and a heterologous viral glycoprotein. Pseudotypes also allow for the study of glycoproteins from highly pathogenic viruses without a BSL3 or BSL4 facility. Virus from different families has been efficiently pseudotyped including SARS coronavirus (SARS-CoV) to compare live virus infection against pseudotypes in a single round of infection, SARS-CoV-2 to test immune response to vaccine candidates, CHIKV to analyse cellular tropism of the GP, Hepatitis C (HCV) to test the influence of the packaging plasmid and the GP ratio on the infectivity of the pseudotypes, Vesicular stomatitis virus (VSV) to alter the tropism of the pseudotypes and infect a wide variety of cells, and Ebola virus (EBOV) to test which mutations had an impact during the West African outbreak [13–19].

## Methods

### Cell culture

HUH7, HEK 293T, CHO, BHK-21 and VeroE6 cells were grown at 37 °C with 5 % CO_2_ in DMEM medium with 1 % NEAA (Non-Essential Amino Acids, Gibco, Thermofisher) and 10 % inactivated FBS (Gibco, Thermofisher), no antibiotics were added to the media. The HEK 293 T cells for transfection were prepared, seeded at 1.5 million cells per dish (Primaria, Corning, Thermofisher) with 10 ml of DMEM media and grown overnight.

### Zika C-PrM-E and C-GFP-PrM-E cloning

Dr Andres Merits kindly provided the template used for the cloning of ZIKV protein sequences. The full-length PrM/E genes and the 3′ 96-nucleotide region of the C gene (corresponding to amino acid residues 101 to 114, which encode the signal peptide) sequence of the two different plasmids (pCCI-SP6-ZIKV-EGFP and pCCI-SP6-ZIKV) were cloned using the primers: forward caccATGCTGAGAATAATCAATGCtagg and reverse CTAAGAGACGGCTGTGGATA. The protocol for the Q5 Hot Start High-Fidelity DNA Polymerase PCR (New England Biolabs) was followed as recommended by the manufacturer (98 °C for 30 s, 25 cycles of 98 °C for 10 s, 63 °C for 20 s, 72 °C for 1 min). The insertion of the foot-and-mouth disease virus 2A protease allows for self-cleavage of the GFP from the C-PrM-E polyprotein.

### Insertion of the product into pcDNA3.1D/V5-His-TOPO

PCR products from the glycoprotein gene were introduced into the vector following the instructions of the manufacturer (ThermoFisher). Briefly, TOPO vector and product were mixed with the salt solution and H_2_O and incubated for 30 min at room temperature (RT). Next, 2 µl of the TOPO cloning reaction was mixed with 50 µl of chemically competent *E. coli* stellar cells and incubated on ice for 30 min. Cells were then heat-shocked at 42 °C for 45 s and incubated on ice for 5 min, after which 250 µl of SOC media was added and incubated on a shaker (200 r.p.m.) for 1 h at 37 °C. Finally, the cells were spread on an ampicillin agar plate, incubated overnight, and the colonies were screened using T7 and BGH primers. The plasmid was purified using the GenElute Plasmid Miniprep kit (Sigma-Aldrich), and quantified using a Nanodrop 1000 (Nanodrop, Thermofisher).

### Pseudotype production

To produce the pseudotypes, HEK 293 T cells were co-transfected with plasmids containing the backbone, luciferase reporter and the glycoprotein. Briefly, for plasmid transfection, we mixed either 2 µg of lentiviral vector pNL4.3.Luc.R(-)E(-) or 2 µg of retroviral vector Murine Leukaemia Virus (MLV) Gag/Pol-encoding packaging construct (phCMV-5349) with 2 µg of luciferase-encoding reporter plasmid (pTG126) and the glycoprotein of interest with Opti-MEM media (ThermoFisher Scientific) with 24 µl of Polyethyleneimine (PEI) to a final volume of 600 µl and incubated for 1 h at RT. The mixture was then added to the HEK 293T seeded dishes with 7 ml of fresh Opti-MEM and incubated for 6 h at 37 °C. The media was replaced with fresh supplemented DMEM (10 % FBS 1 %NEAA), and the cells were incubated for 72 h at 37 °C. Pseudotypes were then harvested and filtered through a 0.22 µm membrane filter and stored at 4 °C for future assays.

### Pseudotype assay using a matrix of concentrations

Different concentrations of each different plasmid were tested to optimize the ratio of the lentiviral vector pNL4.3 [19], the glycoprotein containing plasmid (PrM/M and E) and the NS2B-NS3 ZIKV protease using the protocol for transfection of HEK 293 T cells described above.

### Luciferase assay

HUH7, CHO and BHK-21 cells were seeded in 96-well white plates at 1.5×10^4^ cells per well. HEK 293 T cells were seeded at 1×10^4^ cells per well. The cells were infected with 150 µl of harvested pseudotypes and incubated for 6 h at 37 °C followed by the addition of 150 µl of DMEM media and incubation for 72 h. Cells were lysed using a lysis buffer from luciferase assay system (Promega) following the manufacturer’s instructions, the plate was incubated for 15 min at RT and luminescence was measured using a FLUOstar Omega (BMG LABTECH) plate reader.

### Immunofluorescence (IF)

Briefly HEK 293 T cells were seeded at 50 % confluence on slides for 24 h and transfected with the different plasmids for 72 h, fixed with 2 % paraformaldehyde for 20 min at 4 °C and permeabilised with PermWash solution (PBS 1X, SFB 1 % and saponin 0.2 %) for 20 min at RT. Cells were incubated with the respective primary antibody overnight at 4 °C and with the secondary antibody for 2 h at RT. All antibodies were diluted in PermWash solution, and the nuclei were stained with DAPI. The slides were observed using the Confocal microscope Leica TCS SP8. The images were analysed using the LAS AF lite Software (Leica).

### Western blot

HEK 293T or HUH7 cells were detached from the 10 cm dishes using cold PBS and were centrifuged at 300 ***g*** for 7 min. Pseudotypes were concentrated by ultracentrifugation at 82 000 ***g*** for 1 h 45 min through a 20 % sucrose cushion. The pelleted cells and pseudotypes were then lysed using RIPA buffer adding the protease inhibitor cOmplete (Roche) when needed. The equivalent of 15 µg of protein was loaded into 10 % Mini-PROTEAN TGX precast gels under either reducing or non-reducing conditions. The gels were run for 35 min at 180 volts then transferred onto 0.45 µm PVDF membrane that was previously activated with methanol. The transfer was carried out using the Trans-Blot Turbo system from Bio-Rad using the standard protocol (1.0 A; 25 V constant for 30 min). The membranes were blocked using 10 % Non-Fat-Dried Milk in 1X PBS 0.1 % Tween at RT for 2 h. Primary antibody was incubated overnight at 4 °C followed by consecutive washes with PBS tween 0.1 %, the secondary HRP antibody was incubated for 2 h at RT. The membrane was revealed using the Radiance HRP substrate for CCD imaging (Azure Biosystems), the GBOX chemo XX6 (Syngene) was used as an imaging acquisition system.

### Antibodies

The primary antibodies α-NS3 GTX12452(1 : 200), α-E s4G2 clone Hb119 (1 : 200), and the secondary antibodies rabbit α-mouse AF555 (A-21427, Invitrogen), goat α-rabbit AF594 (A-11012, Invitrogen) were diluted in a PermWash solution. Primary antibodies α-GAPDH (1 : 4000) (A01622, Genescript), α-Flavivirus Glycoprotein (1 : 1500) (Ab214336, Abcam), α-p24 (1 : 1000) (ab9044, Abcam), α-VSV-Glycoprotein (VSV-G) (1 : 1000) (V-5507, Sigma) and secondary α-goat IgG HRP (1 : 4000) (A8919, Sigma), α-mouse immunoglobulins HRP (P0260, Dako) were diluted on 10 % Non-Fat-Dried Milk in 1X PBS 0.1 % Tween.

### Statistical analysis

Data are presented as scatter plots or histograms with mean and sd shown, unless otherwise stated in the figure legends. Comparison of luciferase activity in producer cells were analysed using one-way ANOVA with Dunnett’s multiple comparison test. Significance levels are *P<0.05; **P<0.01; ***P<0.001; ****P<0.0001. Analyses were performed using GraphPAD PRISM 9.1.2

## Results

### Alternative Zika glycoprotein constructs demonstrate no difference in infectivity tests

**Fig. 1.**
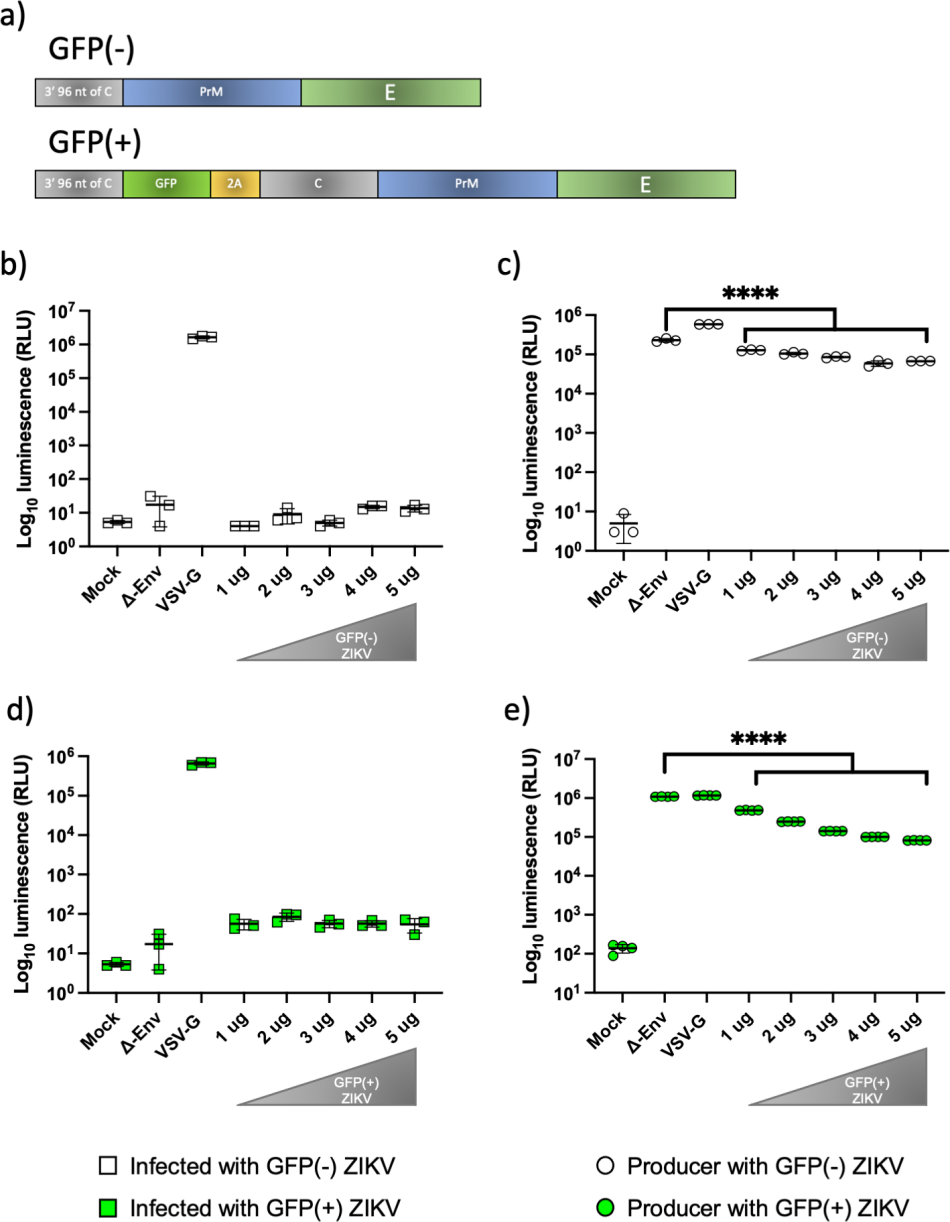
Addition of GFP into the ZIKV GP construct had no effect on measured luciferase activity in both producer and infected cells. (a) Schematic of the two ZIKV glycoprotein constructs tested. Luciferase activity measured in (b) infected and (c) producer cells using the GFP(-) ZIKV construct. Luciferase activity measured in (d) infected and (e) producer cells using the GFP(+) ZIKV construct. VSV-G and Δ envelope were used as positive and negative controls, respectively. Graphs show mean±sd of three independent experiments.

Two constructs of the glycoprotein containing either the 3′ 96-nucleotide region of the C gene (corresponding to amino acid residues 101 to 114, which encode the signal peptide) and full-length PrM/E genes or 3′ 96-nucleotide region of the C gene, GFP, foot-and-mouth disease virus 2A protease and full-length C/PrM/E genes were assessed for their ability to be incorporated into the pseudotypes and influence their infectivity (Fig. 1a). A concentration of 2 µg of pNL4.3 HIV-1 based plasmid was used as the backbone for the pseudotypes, with concentrations between 1 and 5 µg of the ZIKV glycoprotein (GP) tested with both the GFP(-) or GFP(+) constructs. The backbone plasmid without any viral glycoprotein was used as a negative control (∆ Envelope) and VSV-G was used as a positive control. HEK 293 T cells were incubated with the GFP(-) ZIKV pseudotypes to test the infectivity of the particles; no difference was observed between the negative control and the GFP(-) ZIKV pseudotypes (Fig. 1b). Luciferase activity (relative luminescence units, r.l.u.) was also measured in the cells that produced the pseudotypes with a decrease in the enzymatic activity observed when the negative control was compared with the cells supplemented with the GFP(-) ZIKV (Fig. 1c). The GFP(+) ZIKV construct was tested similarly; no difference was observed between the control and the increasing concentrations of GFP(+) ZIKV (Fig. 1d). Measurement of the luciferase activity in the GFP(+) ZIKV producer cells showed a decrease (Fig. 1e) similar to that seen in the GFP(-) ZIKV producer cells (Fig. 1c). As no difference was observed the GFP(+) ZIKV plasmid was used in all subsequent experiments.

### HIV-1, ZIKV and VSV protein expression in producer cells and pelleted pseudotypes

**Fig. 2.**
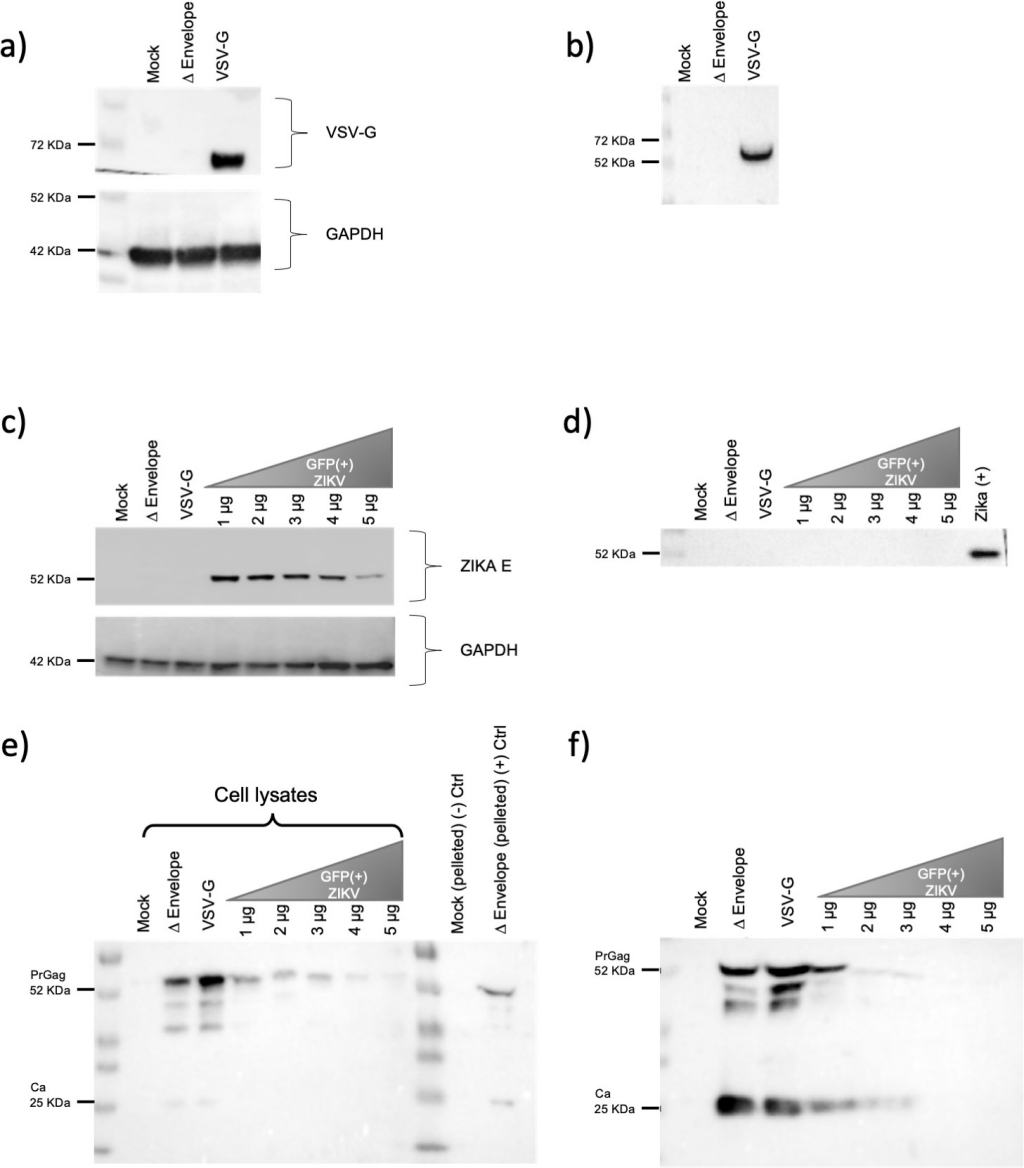
Detection of viral proteins in cell lysates and pelleted pseudotypes by Western blot. The presence of the positive control VSV-G protein was visualized in (a) cell lysates and (b) pelleted pseudotypes. Intensity of ZIKV envelope staining in cell lysates (c) was inversely related to the amount of plasmid transfected. No ZIKV envelope was visible in pelleted pseudotypes (d). Intensity of HIV capsid precursor protein (p55) in cell lysates (e) was inversely related to the amount of GFP(+) ZIKV plasmid transfected. Low levels of processed p24 capsid protein were detected in the pelleted pseudotypes (f).

Western blots against different viral proteins were performed. VSV-G was detected in both the lysates of the producer cells (Fig. 2a) and the pelleted pseudotypes (Fig. 2b), which corresponds with the luciferase activity previously shown (Fig. 1d, e). ZIKV GP expression in the producer cells was detected. However, there is an inverse relationship between the plasmid concentration and protein expression, as the plasmid concentration increases, production of viral GP decreases (Fig. 2c). In Western blots of the pelleted pseudotypes no band was detected for the ZIKV GP at any concentration (Fig. 2d). HIV capsid precursor protein (p55) was detected in the producer cells. However, in the samples where there is a combination of pNL4.3 and ZIKV GP plasmids, there is an apparent reduction of ZIKV GP protein expression (Fig. 2e). For the pelleted pseudotypes, low levels of protein expression was detected for processed p24 capsid protein, which similar to the p55 seems to reduce with higher amounts of ZIKV GP (Fig. 2f).

### Immunofluorescence of HEK 293T cells transfected with the GP and backbone plasmids

**Fig. 3.**
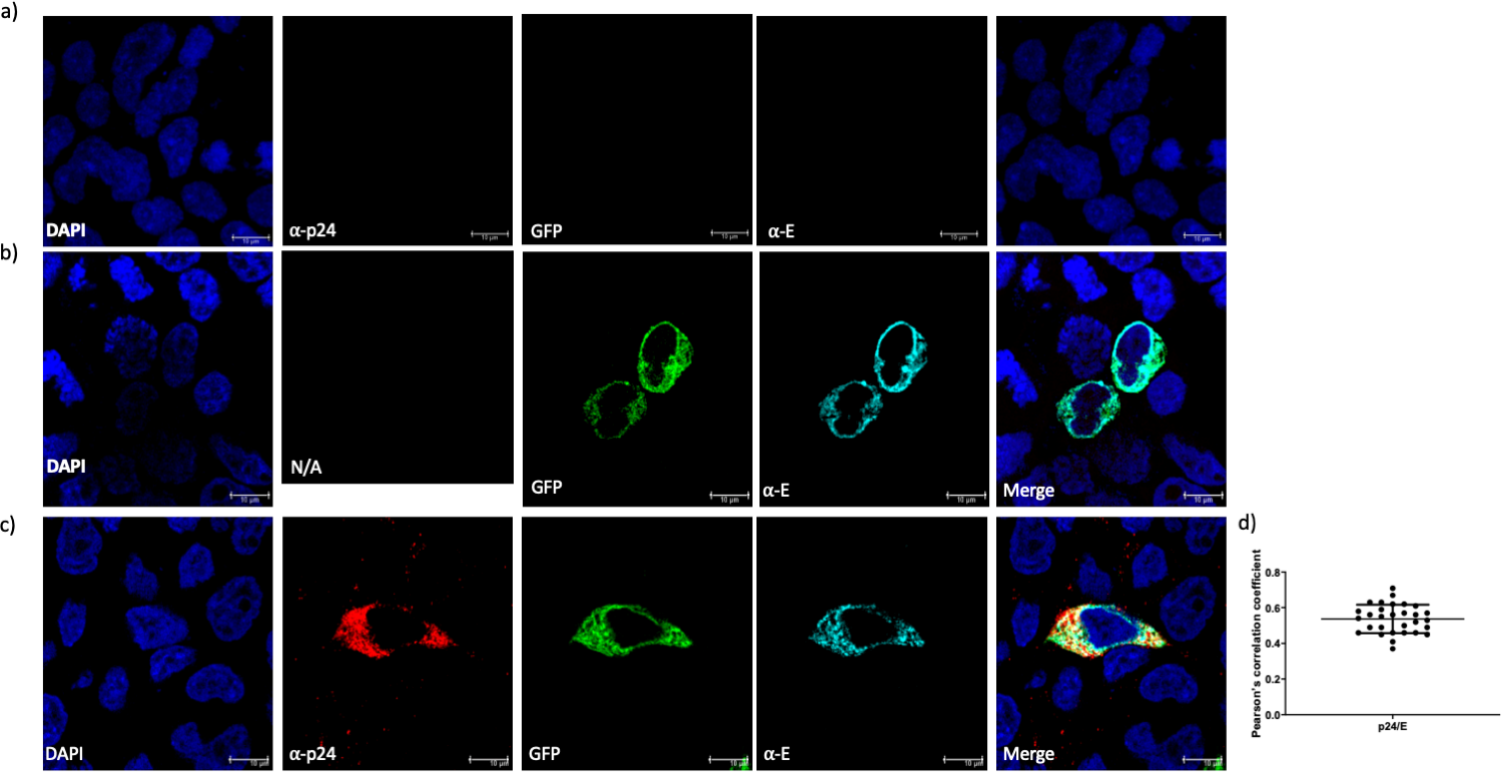
Immunofluorescence of transfected HEK 293 T cells against HIV-1 and ZIKV proteins. (a) HEK 293T control cells showing no cross reactivity or background fluorescence. (b) HEK 293 T cells transfected with the GFP(+) ZIKV glycoprotein plasmid expressed both the reporter protein (GFP, green) and the viral GP/ E (α-E, cyan). (c) HEK 293 T cells transfected with both GFP(+) ZIKV glycoprotein plasmid and pNL4.3 HIV-1 plasmid expressed ZIKV E, and the capsid p24 HIV-1 protein (α-p24, red). (d) Pearson's correlation coefficient graph showing a moderate colocalization between ZIKV E and HIV-1 p24 (*r*=0.53). Scale bar of 10 µm.

Immunofluorescence was performed against the E protein of Zika and the p24 capsid protein of HIV. Cells transfected with the ZIKV GP plasmid and cells transfected with both the ZIKV GP and pNL4.3 plasmid were analysed. HEK 293 T cells mock treated were used as control of fluorescence (Fig. 3a). E protein was detected on transfected cells (α-E, cyan) with the ZIKV GP plasmid (Fig. 3b), with the reporter protein GFP also detected (GFP, green). When HEK 293 T cells were transfected with both the ZIKV GP and the pNL4.3 plasmid, both ZIKV E and HIV p24 (α-p24, red) were detected (Fig. 3c). Finally, when p24 and E were analysed using Pearson's correlation coefficient a moderate colocalization (*r*=0.53) was observed (Fig. 3d).

### Testing different cell lines and ratios between the backbone and glycoprotein plasmids

**Fig. 4.**
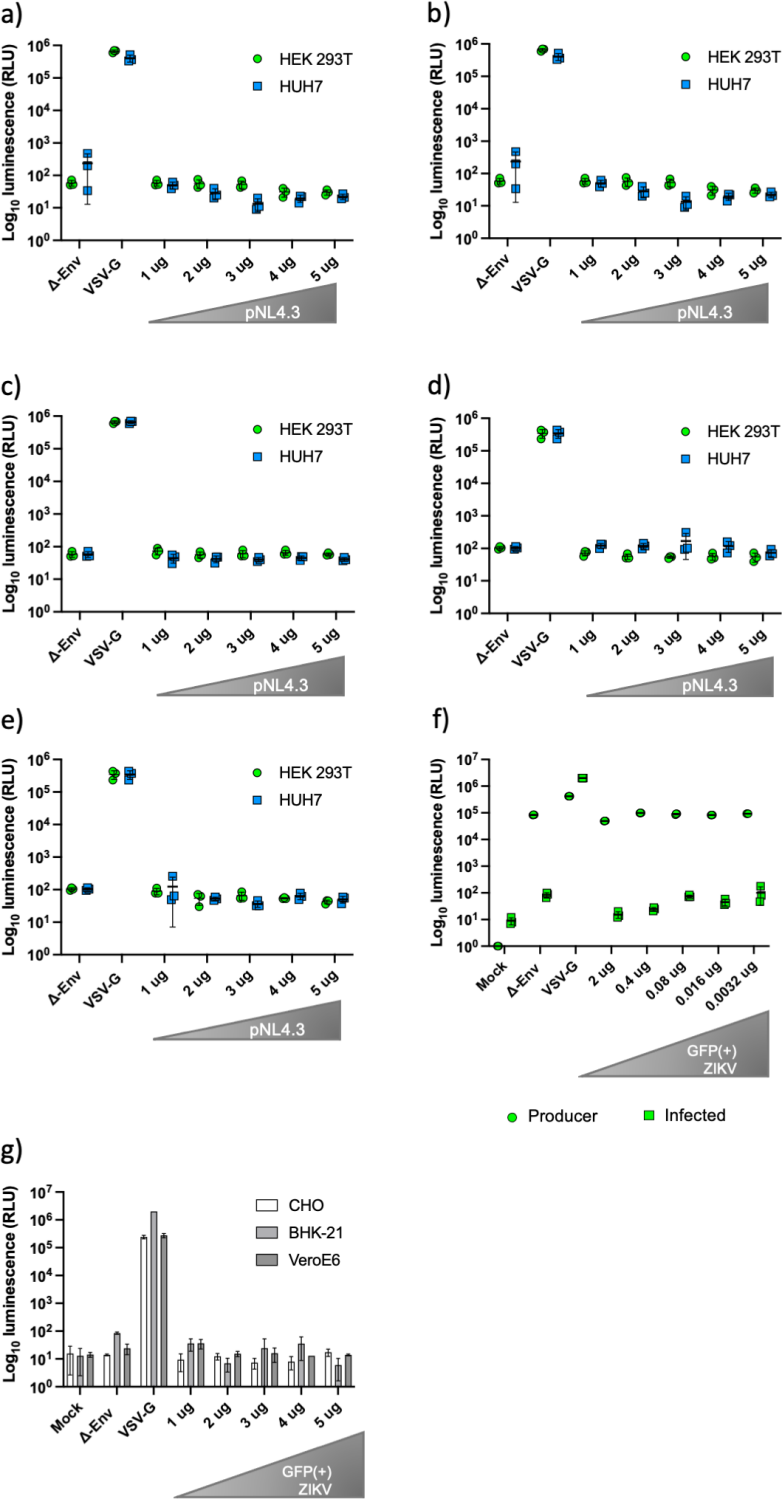
Different cell lines treated with ZIKV pseudotypes. HEK 293T and HUH7 cells infected with pseudotypes produced with different ratios of GFP(+) ZIKV glycoprotein and pNL4.3. (a) 1 µg of GFP(+) ZIKV with a range (1–5 µg) of pNL4.3. (b) 2 µg of GFP(+) ZIKV with a range (1–5 µg) of pNL4.3. (c) 3 µg of GFP(+) ZIKV with a range (1–5 µg) of pNL4.3. (d) 4 µg of GFP(+) ZIKV with a range (1–5 µg) of pNL4.3. (e) 5 µg of GFP(+) ZIKV with a range (1–5 µg) of pNL4.3. Pseudotypes were not infectious in either cell line, regardless of amount of plasmid used. (f) HEK 293 T cells were transfected with a fixed pNL4.3 plasmid amount (2 µg) and fivefold dilutions of the ZIKV GP plasmid (2, 0.4, 0.08, 0.016 and 0.0032 µg). No infectious ZIKV pseudotypes were generated. (g) Mammalian cell lines CHO, BHK-21 and VeroE6 were treated with pseudotypes produced using increasing concentrations of GFP(+) ZIKV plasmid (1–5 µg) in HEK 293 T cells. Again, no infectivity was measured. VSV-G and Δ envelope were used as positive and negative controls, respectively. Graphs show mean±sd of three independent experiments.

A matrix of five different amounts of pNL4.3 plasmid versus five different amounts of GFP(+) ZIKV plasmid was designed to establish if the lack of infectivity was a result of an imbalance in the backbone to GP ratio. HEK 293T and HUH7 cells were used to test the infectivity of these pseudotypes. A range of 1, 2, 3, 4 and 5 µg of each plasmid were used. Each graph shows a fixed amount of the GFP(+) ZIKV with increasing amounts of the backbone (Fig. 4a–e). No differences were observed on luciferase activity when comparing the measurements of the matrix compared with the Δ-envelope control in either cell type. As previous shown by our lab [19], reducing the amount of glycoprotein plasmid can rescue non-infectious pseudotypes. Using a fixed amount of pNL4.3 (2 µg) and a range of 2, 0.4, 0.08, 0.016 and 0.0032 µg of the GFP(+) ZIKV plasmid, luciferase activity was measured in HEK 293 T cells. No difference was measured compared to the Δ-envelope control (Fig. 4f). Different mammalian cell lines (BHK-21, CHO and Vero E6) were used to confirm the lack of infectivity, using the same conditions as the initial HEK 293T experiment with 2 µg pNL4.3 and a range of 1–5 µg GFP(+) ZIKV plasmid (Fig. 4g).

### Supplementation with ZIKV NS2B-NS3 protease and substitution of the backbone

**Fig. 5.**
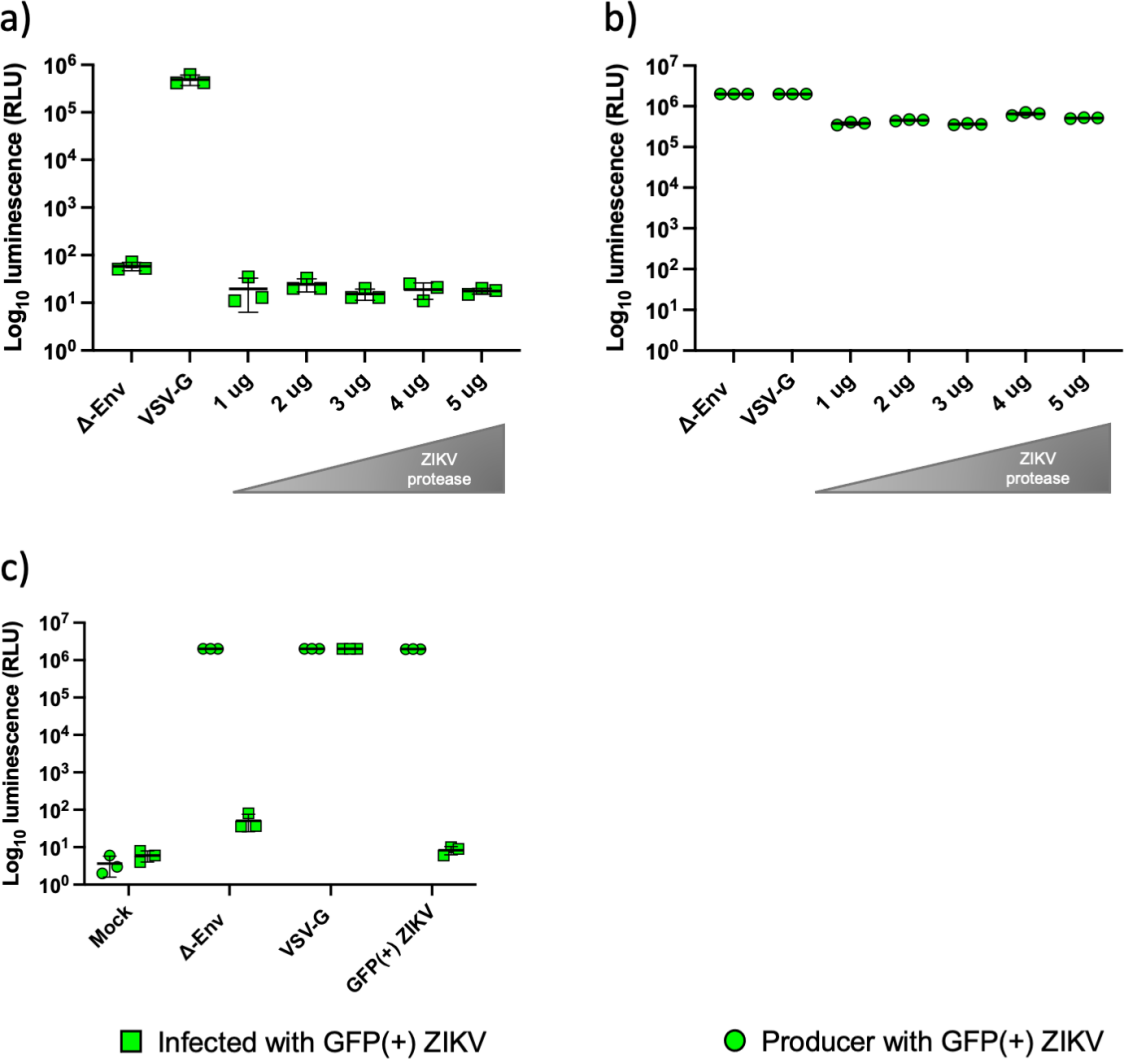
Luciferase activity measured in the producer and infected cells supplemented with viral protease plasmid. HEK 293 T cells were co-transfected with 2 µg of pNL4.3 and 2 µg of GFP(+) ZIKV. Increasing amounts (1–5 µg) of the NS2B/NS3 protease plasmid were added. Luciferase activity measured in (a) infected and (b) producer cells showed no infectious pseudotypes produced and no reduction in luciferase activity in the producer cells. (c) Murine Leukaemia Virus (MLV) backbone was tested at 2 µg with 2 µg GFP(+) ZIKV to identify if the backbone had an effect on infectivity. No infectious particles were produced. VSV-G and Δ envelope were used as positive and negative controls, respectively. Graphs show mean±sd of three independent experiments.

To confirm that the GP was undergoing correct cleavage the ZIKV NS2B-NS3 protease was added as a plasmid at different amounts to the producer cells. However, this did not increase the infectivity of the pseudotypes (Fig. 5a). No reduction in luciferase activity was measured in the producer cells (Fig. 5b). To investigate the possibility of the backbone being incompatible with this glycoprotein, the lentiviral pNL4.3 HIV-1 backbone was substituted with a retroviral MLV backbone, however no difference was observed between the Δ-envelope control and the GFP(+) ZIKV pseudotypes when tested at 2 µg of each plasmid (Fig. 5c). Further testing with different amounts of MLV backbone plasmid did not produce infectious pseudotypes (data not shown)

## Discussion

ZIKV became a public health problem on the American continent beginning in 2015 and continuing into 2016, following the pattern of other arboviruses such as CHIKV and DENV [20]. During the progression of the ZIKV epidemic across Brazil, CZS appeared to impair the neural development in children, further raising the alarm of significant long-term health impacts [21]. The initial evidence for the cellular receptor for ZIKV pointed to members of the TAM family of receptor tyrosine kinases (RTKs); Tyro3, Axl and Mertk being essential for infectivity, with Axl being the key player. Axl has been reported to be expressed on cortical astrocytes, microglia and progenitors in the neural retina. Interestingly when mouse and ferret models were used, Axl expression in radial glia neural stem cells was conserved as observed in human induced pluripotent stem cells (iPSC)-derived cerebral organoids [11]. ZIKV was also able to infect human testicular organoids (HTO), which highly express Axl, and endothelial cells are also susceptible to ZIKV infection, which also express Axl [22, 23]. However later reports showed that *Axl-*deficient mice were able to sustain viral replication with no difference to *Axl* positive mice, other experiments showed that removal of *Axl* does not protect against ZIKV in 2D NPC culture or 3D cerebral- organoids [23, 24]. When Tyro3 and Mertk were studied, no difference was found on viral load in the knockout (*Axl-/-/Tyro3-/-, and Axl-/-/Mertk-/-*) or wild type mice infected intracranially [25]. These mixed reports highlight the opportunity of using different strategies to identify susceptible cell lines and possible cellular receptors.

Pseudotypes provide an excellent option, due to their flexibility and safety. For example, Ebola pseudotypes based on an MLV backbone have been used to identify susceptible or resistant cell lines, and Rubella virus GP was pseudotyped using the VSV system to demonstrate non-immune cells were generally susceptible, whilst immune cells were much less susceptible [26, 27]. Designing a pseudotype assay for the *Flaviviridae* has been elusive, with success seen only amongst the hepacivirus genus, most notable with HCV [19, 28]. In this study we investigated methods to produce infectious pseudotypes using ZIKV GP, following a previously reported design, where the signal peptidase signal peptide of C protein was conserved [29]. Two alternative plasmid constructs: one containing full-length PrM/E genes and the 3′ 96-nucleotide region of the C gene (corresponding to amino acid residues 101 to 114, which encode the signal peptide) and the other containing ZIKV proteins plus a GFP reporter gene to track the expression of the glycoprotein were constructed. Initially, general pseudotyping parameters were optimised using a fixed amount of the pNL4.3 backbone (2 µg) and increasing concentrations of the GP containing plasmids [GFP (+) or GFP (-)] [19]. HEK 293 T cells were selected as a model for the infectivity assay due to previous reports of their susceptibility to ZIKV infection [30, 31]. No difference was observed between the luciferase activity of the ∆ Envelope negative control and the cells treated with the pseudotypes produced with increasing concentrations (1 to 5 µg) of either the Zika PrM/E GFP (+) or GFP (-) plasmid (Fig. 1b, c). When the enzymatic activity was measured in the producer cells an interesting phenomenon was observed, luciferase activity was inversely correlated to the concentration of the GP containing plasmid. This effect was conserved for both the GFP (+) or GFP (-) version of the plasmid and there was a significative difference (*P*=0.0001) with the negative control with values of the RLU falling under the basal activity of the ∆ Envelope producer cells. These results suggest that the co-transfection of pNL4.3 backbone and ZIKV GP plasmid could interfere in some way with the proper expression of the viral proteins, this observation is supported by the measurements of the VSV-G positive control that showed similar levels of luciferase activity in both the producer and the infected cells.

We then assessed expression of viral proteins in both the producer cells and the pellets of the pseudotypes to determine if the phenomenon observed in the luciferase assays was mirrored in other proteins supplemented on the plasmids. VSV-G protein was observed in both the producer cells and the pelleted pseudotypes by Western blot analysis (Fig. 2a, b). However, when ZIKV E was blotted, similar results were observed with less intense bands as the amount of the ZIKV PrM/E increased (Fig. 2d), no signal were detected on the pelleted supernatants (Fig. 2d). These results explain why there was no difference in luciferase activity above the negative control on the infected cells since there was no ZIKV glycoprotein incorporated to the pseudotypes.

Finally, HIV-1 p55 capsid precursor protein expression was investigated, there was a noticeable decrease of protein expression in the producer cells supplemented with the ZIKV GP plasmid compared with the negative and positive control (Fig. 2e). HIV-1 capsid p24 showed the same pattern when the pelleted pseudotypes were blotted (Fig. 2f). HEK 293 T cells transfected with the ZIKV GP and HIV backbone were analysed using IF, all conditions showed expression of the viral proteins (Fig. 3b, c), however when colocalization was measured (Fig. 3d) only a moderate value was obtained, this suggest that the interaction between the capsid and matrix protein of HIV and the ZIKV glycoprotein, needed to form the pseudotypes, may not be present.

Comparing the results from the luciferase assays and the Western blots, there is a clear relationship between the expression of pNL4.3 HIV-1 and ZIKV GP proteins and the luciferase activity, as we increased the amount of GP plasmid less protein was detected for both viruses and less enzymatic activity. We therefore hypothesized based on previous reports [19, 32] that the plasmid ratios could be the determinant factor for a successful pseudotype production. A matrix of 25 different plasmid ratios was designed to try to improve infective pseudotype production, a range of 1 to 5 µg of both the backbone and the glycoprotein was tested (Fig. 4a). No difference in the r.l.u. was observed between the negative control and the samples corresponding to the matrix when HEK 293T or HUH7 cells were treated with the pseudotypes suggesting that none of the different ratios tested had any positive impact in the infectivity. To test the possibility that the HEK 293T and HUH7 were refractive to the infection, BHK-21 and VeroE6 cells were used, as previous reports have shown them to be susceptible and CHO cells were used as a line that is resistant to the infection (Fig. 4b). No difference in luciferase activity was observed between the negative control and the ZIKV pseudotypes in any of the cell lines [33, 34]. Three other alternative methods were tested to produce pseudotypes; dilution of the GFP(+) ZIKV plasmid, addition of a viral protease and changing the lentiviral backbone for a retroviral MLV. The fivefold dilutions of the GFP(+) ZIKV plasmid (2, 0.4, 0.08, 0.016 and 0.0032 µg) with a fixed amount of pNL4.3 backbone (Fig. 4f) showed no difference between the negative control and the pseudotypes produced with the fivefold dilution. Previous reports have shown that supplementation with viral proteases enhances pseudotype production [35–37]. A tri-plasmid system composed of a fixed concentration of GFP(+) ZIKV and pNL4.3 and increasing amounts of ZIKV NS2B-NS3 containing plasmid was tested (Fig. 5b). Plasmid supplementation of the protease was carried out to mimic the natural life cycle of the virus. The protease is located on different cellular locations such as the cytoplasm and replicative complexes where it cleaves the viral polyprotein at different sites. It plays an important role in the processing of prM protein by cleaving the junction between C-prM thus releasing the prM/E proteins from the capsid [38, 39]. The addition of the viral protease did not have any impact on the infectivity, suggesting that the protein was properly processed as suggested by Western blot and immunofluorescence. In contrast with other cases where supplementation with the purified protease, such as furin, improves glycoprotein processing, this strategy may not be effective for NS2/NS3 as the C-prM cleaving event occurs prior to viral assembly and particles with fused C-prM are not formed. Finally, MLV backbone was evaluated using 2 µg of GFP(+) ZIKV and 2 µg of MLV (Fig. 5c), this substitution did not have any impact in the infectivity either.

Several phenomena may explain the incapacity of the producer cells to incorporate the ZIKV GP into the pseudotypes and infect cell lines. Four mechanisms describe the incorporation of the GP to the pseudotype (A) random interaction, (B) similar targeting, (C) direct interaction, (D) indirect interaction. Direct interaction between the gag protein and the cytoplasmic tail (CT) of ZIKV E could be essential to proper protein packing, a similar phenomenon was observed in Rous sarcoma virus (RSV) [40]. HIV typically buds from the plasma membrane (PM), so the expression of the GP on the PM may be a determinant of proper incorporation. HCV being a member of the *Flaviviridae* family with similar glycoprotein structure showed cellular membrane expression with some level of endoplasmic reticulum retention (ERR) [41]. Modifications to the viral GP have been effectively used to increase the incorporation of the protein into pseudotypes and increasing the infectivity titre by either altering the CT or transmembrane sequence (TM). Truncations in the CT of the SARS-CoV S protein have an impact on the infectivity by either altering incorporation of the GP into the pseudotype, receptor binding or fusion/entry [15]. Other strategies include the construction of chimeric proteins that contain the CT of a protein known to efficiently pseudotype. Substitution of the rabies virus glycoprotein (RVG) CT sequence with that of VSV-G resulted in increased incorporation of the GP into the pseudotype and increased infectivity [42]. Modifying the cytoplasmic tail of the ZIKV GP may enhance the incorporation to the lentiviral backbone thus increasing the infectivity of the particle by either modifying the interaction between the capsid and the glycoprotein or by targeting the ZIKV GP to the appropriate cellular compartment (reducing ERR and targeting to the plasma membrane).

Various attempts had been made to pseudotype members of the *Flaviviridae* family with limited success. As previously mentioned, the main potential obstacle is the different cellular localization of the heterologous glycoproteins and that of the backbone. Similar to lentiviral backbones, VSV pseudotyping has previously been used to generate infectious particles of a wide variety of viruses such as Ebola virus, Hepatitis C virus and Japanese Encephalitis virus (JEV) [43–45]. To generate the VSV-based pseudotypes, cells are transfected with a plasmid containing the heterologous glycoprotein and infected with a VSV G-complemented pseudotype virus (*G-VSVΔG). The supernatant from the producer cells is then harvested and used to infect naïve cells with the infectivity measured using a reporter gene such as luciferase or GFP [45]. As an alternative to this system of transfection/infection, other groups have developed cell lines with self-replicating subgenomic replicons that produce non-structural viral proteins, structural flaviviral proteins and the packaging system were then supplemented on separate plasmids. This strategy was used on other flaviviruses like DENV and WNV with positive results [46–48]. However, due to the multiple steps and complexity required to an efficient production of infectious particles either by VSV or replicon systems, our scope was to develop a simple method to produce infectious pseudotypes based on lenti- or retro-viruses. Recent evidence showed that ZIKV pseudotypes could be produced using higher amounts of the glycoprotein plasmid (8 µg) and the retroviral backbone pNL Luc AM Nef ^+^ (37 µg). According to the data obtained from these experiments, this amount of plasmid had a positive impact on the glycoprotein incorporation and particle formation; the substitution of the pNL4.3 backbone by pNL Luc AM Nef ^+^ directly impacted on the cytoplasmic delivery of the HIV-1 genome improving the pseudotype production and infectivity [49].

Altogether, the information presented here shows that the strategy followed in this paper to pseudotype Zika PrM/M/E glycoprotein using pNL4.3 and MLV backbones does not produce infectious pseudotypes. Proteins from both the backbone and the heterologous GP are being produced; however, they are not correctly incorporated into infectious particles. Further modifications to the GP cytoplasmic tail or the addition of other viral accessory proteins may benefit the incorporation. Nevertheless, the impact of these modifications on the protein structure and function must be evaluated.
